# Evaluation of a high-throughput *Candidozyma auris* assay for use on the cobas 5800/6800/8800 omni Utility Channel

**DOI:** 10.1128/spectrum.00254-25

**Published:** 2025-05-16

**Authors:** Jeffrey Alexander, Natacha Martins-Sorenson, Arrash Moghaddasi, Andrew T. Hill, Jody Harris, Stephen McCune, Erin Gick, YanChun Zhu, Sudha Chaturvedi, Michael Lewinski, Kyle C. Cady

**Affiliations:** 1 Roche Molecular Systems 53527 , Pleasanton, California, USA; 2 Roche Molecular Systems, Santa Clara, California, USA; 3 Roche Diagnostics Corporation 43560 , Indianapolis, Indiana, USA; 4 Mycology Laboratory, Wadsworth Center, New York State Department of Health 375448, Albany, New York, USA; Central Texas Veterans Health Care System, Temple, Texas, USA

**Keywords:** *Candidozyma*, diagnostics, hospital infections

## Abstract

*Candidozyma auris* (formerly known as *Candida auris*) is an emerging worldwide fungal pathogen associated with difficult-to-treat nosocomial infections. Screening of patients for colonization is a recommended strategy to track and prevent hospital spread and outbreaks caused by *C. auris*. To facilitate this, a high-throughput (HTP) assay with a short turnaround time and minimal manual processing has significant value. Here, we present ongoing research on the detection of *C. auris* by direct testing of nasal/axilla/groin swab samples using the HTP cobas 5800/6800/8800 family of systems that has the ability to process up to 1,056 samples in an 8 h shift. Based on testing of 200 surveillance swab samples, the cobas *C. auris* Utility Channel (UC) assay demonstrated 94% accuracy, 91% positive percent agreement, and 98% negative percent agreement when compared to an established nucleic acid amplification test (NAAT) (BD MAX Open System assay). In a secondary analysis of 159 out of the 200 samples, the assay was compared to standard-of-care culture results with an observed 95% accuracy, 92% sensitivity, and 90% specificity. The cobas *C. auris* UC assay was inclusive for clades I–V and exclusive to 15 other closely related fungal species based on wet lab testing. The data presented originated from internally conducted studies performed for research purposes. The cobas *C. auris* UC assay is not approved for *in vitro* diagnostic use.

**IMPORTANCE:**

*C. auris* is an emerging multidrug-resistant fungal pathogen and considered a global public health threat. Rapid detection and identification of *C. auris* is critical for hospital infection prevention and control and outbreak surveillance. The standard-of-care testing for *C. auris* involves culture-based methods with a long turnaround time. A rapid assay with higher throughput and quicker turnaround time can facilitate measures to minimize potential spread and prevent outbreaks within healthcare facilities. In the internal research study presented here, we evaluated a fully automated HTP *C. auris* assay for direct testing of nasal/axilla/groin swab samples on a commercially available platform and compared the results to an established NAAT assay.

## INTRODUCTION

*Candidozyma auris* is an ascomycetous budding yeast that has simultaneously evolved across the world and is a known skin-colonizing microbe which can be confused with similar fungal organisms, such as *Candidozyma haemuli* (formerly *Candida haemulonii*), *Candidozyma duobushaemuli* (formerly *Candida duobushaemulonii*), and *Rhodotorula glutinis*, when utilizing phenotypic methods (1–3). This simultaneous evolution has led to five distinct clades contributing to hospital outbreaks worldwide (4–6), with a new sixth clade recently identified in Singapore (7). *C. auris* has a high rate of multidrug resistance, causing outbreaks of invasive disease with significant morbidity and mortality in regions where it has become established (5, 8, 9). Due to the high in-hospital mortality rates, multidrug resistance, and increased hospital spread, *C. auris* is considered an urgent health threat by the Centers for Disease Control and Prevention (CDC) and a fungal pathogen of critical priority by the World Health Organization (10, 11).

Treatment of *C. auris* infections can be challenging due to its high level of drug resistance to the three available classes of antifungals: azoles, polyenes, and echinocandins (1). Patients who have weakened immune systems or with longer stays in intensive care units are at the highest risk of infection, with mortality rates of 30%–60% (12–14). Once a patient is colonized, *C. auris* can transfer easily from patient to patient and to the hospital environment, surviving up to a week and causing outbreaks (15, 16). Implementation of rapid *C. auris* testing at hospital admission can enable infection prevention/control practices and decrease transmission, especially in high-risk settings (17, 18).

Misidentification of *C. auris* and slow turnaround times using conventional culture-based and/or yeast identification systems such as the VITEK 2 YST (BioMérieux), API 20°C (BioMérieux), BD Phoenix yeast (Becton Dickinson), and MicroScan (Beckman Coulter) (19–21) are long-standing challenges. Similarly, matrix-assisted laser desorption ionization time-of-flight (MALDI-TOF) mass spectrometry as a standard identification method for *C. auris* relies on the growth of pure isolates that can take from 4 to 14 days (22). Molecular testing can overcome these shortcomings with high sensitivity and specificity assays run directly from patient samples. This research work aimed to evaluate a fully automated high-throughput (HTP) assay utilizing the cobas 5800/6800/8800 omni Utility Channel (UC).

## RESULTS

**Fig 1:**
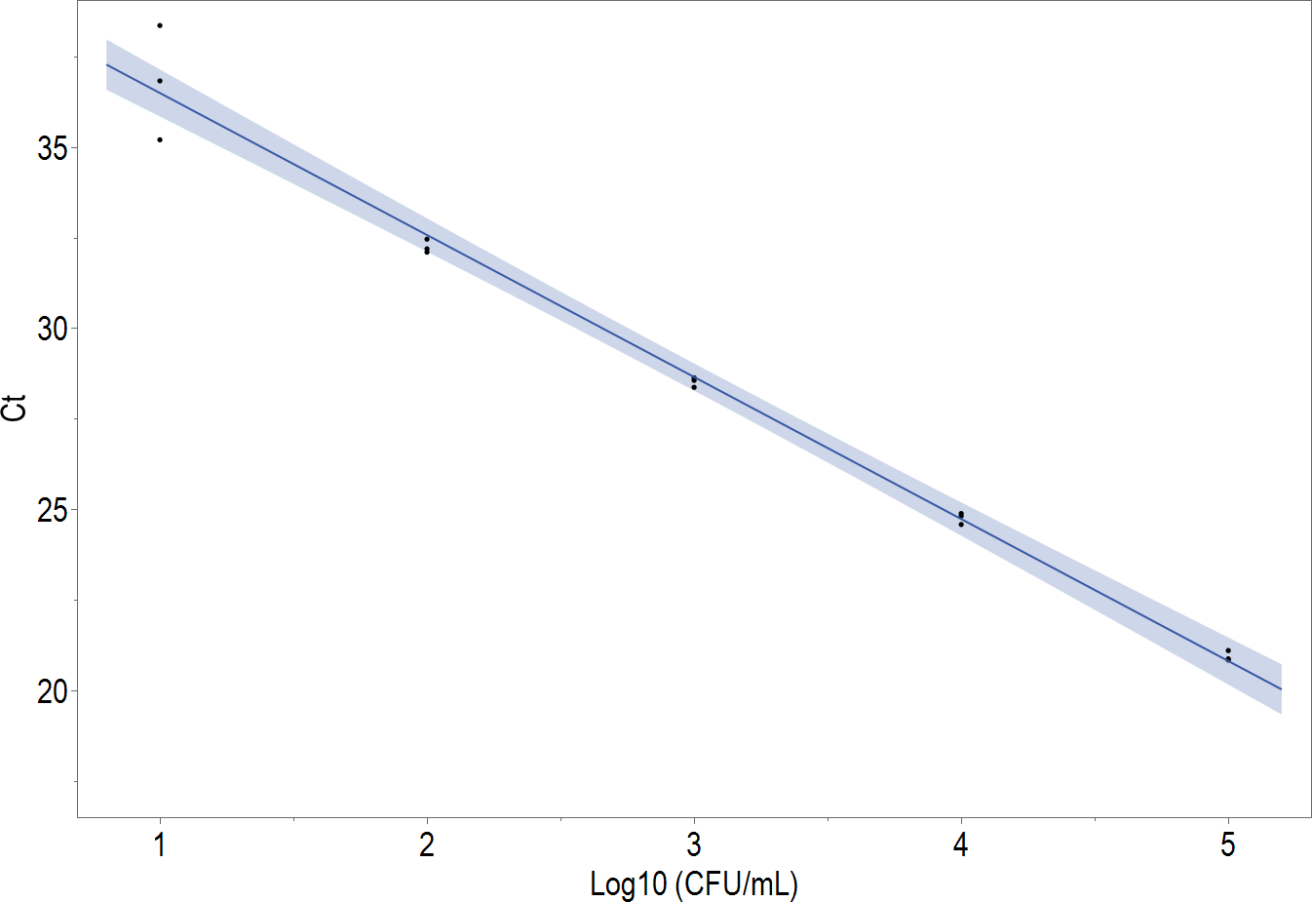
Linearity of cobas *C. auris* UC assay utilizing the Zeptometrix Z485 cell line. The samples were tested in triplicate from 10^5^ CFU/mL down to 10 CFU/mL, *R*^2^ = 0.99. CFU, colony-forming unit; Ct, cycle threshold.

**Fig 2:**
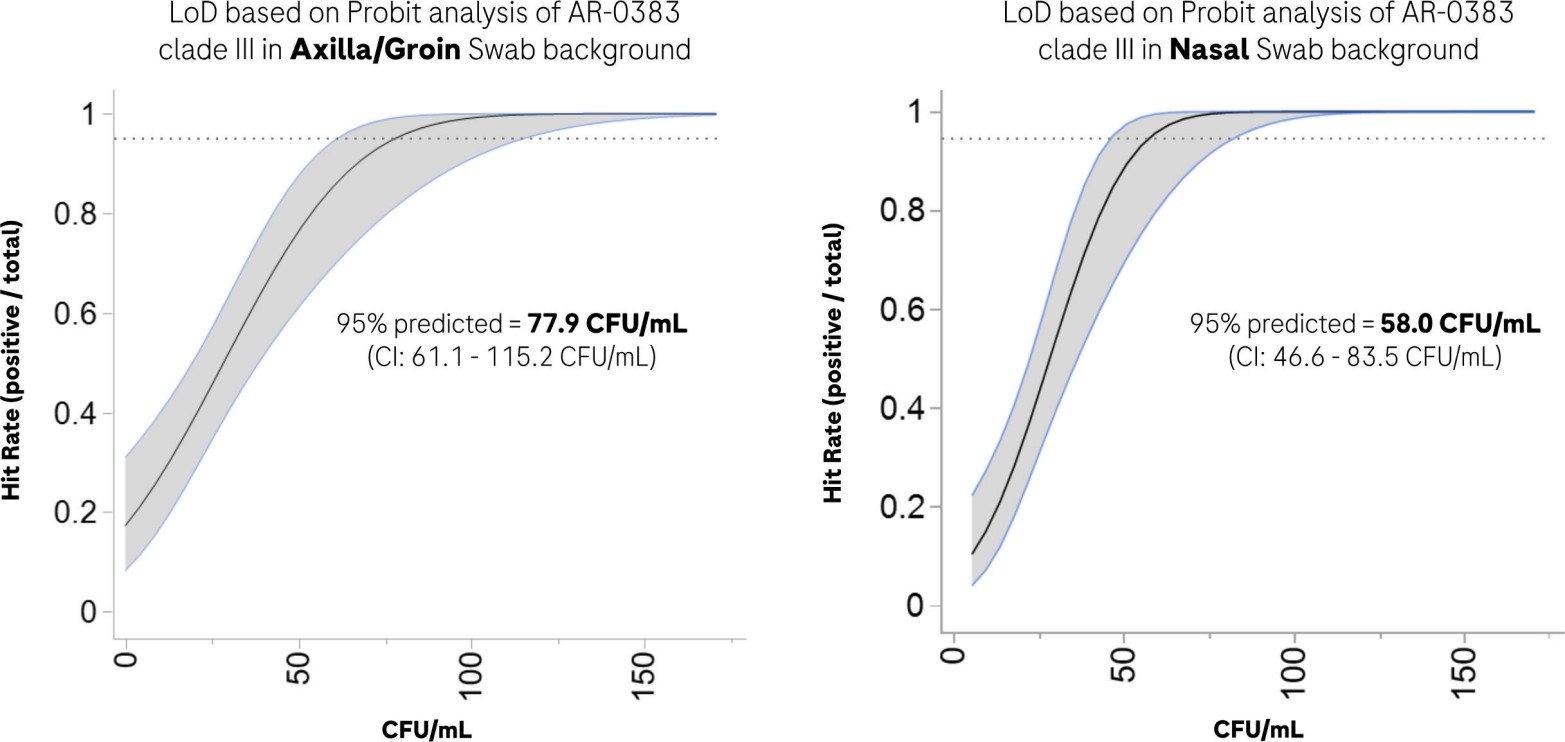
Limit of detection of AR-0383 clade III cell line in two different human backgrounds (axilla/groin and nasal swab) in cobas PCR collection media. Probit analysis with 95% ± confidence interval (shaded region) for each collection site.

As *C. auris* is known to have six diverse clades, we confirmed that the assay oligos are fully inclusive to all clades based on *in silico* analysis (736 available National Center for Biotechnology Information [NCBI] *C. auris* sequences). Wet lab testing was limited to clades I–V CDC strains as clade VI was not yet available for testing because it was only recently discovered. However, the assay’s 5.8S ITS.2 target sequence remains conserved within this subtype, supporting *C. auris* inclusivity (data not shown). Conversely, no cross-reactivity was observed for the 15 closely related fungal species tested, including *C. haemulonii*, *C. duobushaemulonii*, *Candida sake*, and *Candida famata* (Table 1). No false-positive signals were observed when screening 200 blank samples of both liquid Amies and cobas PCR Media (CPM) (data not shown). Assay linearity was also observed across five orders of magnitude with *R*^2^ = 0.99 (Fig. 1). Limits of detection (LoDs) of 77.9 and 58.0 CFU/mL were determined using *C. auris-*spiked samples in axilla/groin and nasal backgrounds, respectively (Fig. 2).

**TABLE 1:** Exclusivity testing of the cobas *C. auris* UC assay with 15 closely related *Candida* spp. in duplicate at 10^7^ CFU/mL^*a*^

Species	Source	Source ID	Channel 3 (*C. auris* target)		
			Avg Ct	Avg RFI^*c*^	Overall result call
*Candida albicans*	ATCC	38289	NaN^*b*^	1	Negative
*Candida albicans*	ATCC	18804	NaN	1	Negative
*Candida dubliniensis*	USDA	NRRLY-17841	NaN	1	Negative
*Candidozyma duobushaemuli*	CDC	AR-0391	NaN	1	Negative
*Candidozyma duobushaemuli*	CDC	AR-0394	NaN	1	Negative
*Candidozyma duobushaemuli*	CDC	AR-0392	NaN	1	Negative
*Candida famata*	ATCC	201067	NaN	1	Negative
*Candida famata*	ATCC	60229	NaN	1	Negative
*Candida glabrata*	ATCC	2001	NaN	1	Negative
*Candida glabrata*	ATCC	MYA-2950	NaN	1	Negative
*Candida guilliermondii*	ATCC	14242	NaN	1	Negative
*Candidozyma haemuli*	ATCC	22991	NaN	1	Negative
*Candidozyma haemuli*	CDC	AR-0392	NaN	1	Negative
*Candidozyma haemuli*	CDC	AR-0393	NaN	1	Negative
*Candida kefyr*	ATCC	34137	NaN	1	Negative
*Candida krusei*	ATCC	14243	NaN	1	Negative
*Candida krusei*	CDC	AR-0397	NaN	1	Negative
*Candida lusitaniae*	ATCC	34449	NaN	1	Negative
*Candida metapsilosis*	ATCC	10232	NaN	1	Negative
*Candida orthopsilosis*	ATCC	96139	NaN	1	Negative
*Candida parapsilosis*	ATCC	22019	NaN	1	Negative
*Candida parapsilosis*	ATCC	90018	NaN	1	Negative
*Candida sake*	ATCC	28136	NaN	1	Negative
*Candida sake*	ATCC	22021	NaN	1	Negative
*Candida tropicalis*	ATCC	13803	NaN	1	Negative
*Candidozyma auris*	CDC	AR-0383	24	10	Positive

^*a*^ All exclusive cell lines tested returned a negative result. *C. auris* CDC AR-383 strain tested at 10^4^ CFU/mL returned a positive result as expected.

^*b*^ NaN denotes “not a number” as no Ct value was found, and an RFI average of 1 is baseline.

^*c*^ RFI, relative fluorescence intensity.

The primary analysis of this internal study for research purposes was the testing of 200 remnant clinical swab samples of the nares/axilla/groin, which demonstrated 94% (95% confidence interval [CI]: 90.4–96.9) overall agreement, 91% (95% CI: 83.6–95.1) positive percent agreement, and 98% (95% CI: 93.0–99.4) negative percent agreement when compared to the nucleic acid amplification test (NAAT) reference method (BD MAX Open System workflow) (23) (Table 2). A single sample called positive by the BD MAX (Becton, Dickinson and Company, Franklin Lakes, NJ) workflow had an invalid cobas *C. auris* UC assay result due to insufficient sample volume when received. Of the remaining 11 discrepant samples (2 positive by cobas *C. auris* UC assay and 9 positive by BD MAX), only 1 sample was confirmed positive by the culture enrichment workflow (Table S1). A secondary analysis was performed using 159 samples with available identification by culture to compare NAAT results against culture as the reference method (Table 3). Both assays had a high sensitivity of 100% (95% CI: 95.8–100.0) and 99% (95% CI: 93.7–99.8) for the BD MAX and the cobas *C. auris* UC assay, respectively. However, a lower specificity of 82% (95% CI: 71.5–89.1) was observed for the BD MAX workflow in comparison to the cobas *C. auris* UC with 90% (95% CI: 81.3–95.2) (Table 3).

**TABLE 2:** Comparison of clinical performance from 200 *C*. *auris* surveillance samples between the BD MAX workflow and the cobas *C. auris* UC assay^*a*^

		BD MAX workflow		Overall % agreement (95% CI)	Positive % agreement (95% CI)	Negative % agreement (95% CI)
		# Positive	# Negative			
cobas *C. auris* UC assay	# Positive	90	2	94 (90.4–96.9)	91 (83.6–95.1)	98 (93.0–99.4)
	# Negative	9	98			
	# Invalid	1	0			

^*a*^ CI, confidence interval.

**TABLE 3:** Results from 159 clinical samples processed with both NAAT workflows and how their results compare to culture-based MALDI-TOF ID^*a*^

		Culture enrichment results		Accuracy % (95% CI)	Sensitivity % (95% CI)	Specificity % (95% CI)
		# Positive	# Negative			
BD MAX workflow results	# Positive	87	13	92 (86.5–95.2)	100 (95.8–100)	82 (71.5–89.1)
	# Negative	0	59			
cobas *C. auris* UC assay results	# Positive	85	7	95 (90.3–97.4)	99 (93.7–99.8)	90 (81.3–95.2)
	# Negative	1	65			
	# Invalid	1	0			

^*a*^ CI, confidence interval.

## DISCUSSION

**Fig 3:**
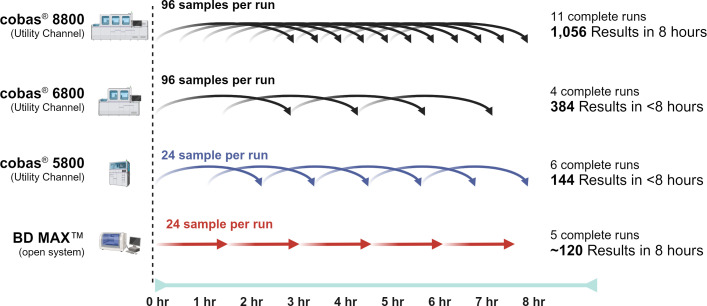
The cobas *C. auris* UC assay, when paired with a cobas x800 instrument, has much higher throughput when compared to the CDC test adapted to the BD MAX workflow.

Here, we performed an internal research study for a HTP *C. auris* assay utilizing the Utility Channel of the cobas 5800/6800/8800 systems. This fully automated sample-in results-out assay has a roughly 3 h turnaround time and can process up to 1,056 samples in an 8 h shift (Fig. 3). These research data support the ability of this assay to be utilized without a pre-analytic step directly on nasal/axilla/groin swab samples. Further studies with analytical and clinical validation are needed to characterize the performance of this assay as a potential laboratory-developed test (LDT).

Based on the described results, the estimated LoD of 50–80 CFU/mL was similar to other manual LDT intended for screening nasal/axilla/groin swab samples (24–26). Our primary analysis comparing the cobas *C. auris* UC assay results to a NAAT reference method (BD MAX workflow) (23) returned comparable performance. However, this internal research study has several limitations when processing clinical samples, including the limited volume (200 µL) and the longer-than-recommended storage conditions for samples used for the cobas *C. auris* UC assay (frozen or fresh), both of which could have impacted performance and contributed to the discrepant results. This includes three samples (Frozen18, Fresh95, and Fresh100) testing negative with the cobas *C. auris* UC assay and a late cycle threshold (Ct) (Ct>36) when tested immediately with the BD MAX workflow (Table S1).

In the secondary analysis comparison with the culture enrichment workflow, a greater specificity was observed with the cobas *C. auris* UC assay versus the BD MAX workflow. The current gold standard for culture enrichment-based identification of *C. auris* is manual and has a longer turnaround time than NAAT assays, making surveillance with this method suboptimal. In addition, NAAT tests can also have increased sensitivity and lower specificity when compared to culture methods (27–29). This increased sensitivity means that a NAAT is more likely to return a positive result than culture methods when the colonization is present at a very low level.

As *C. auris* continues to be a nationally notifiable disease by the CDC and cases continue to rise across the United States (30), the availability of an HTP NAAT capable of being used on established cobas systems could improve testing workflows and enable hospital infection prevention/control practices. In conclusion, this internal research evaluation of an HTP *C. auris* assay on the UC of cobas 5800/6800/8800 systems, using nasal/axilla/groin swabs and multiple collection media (cobas PCR media and liquid Amies), demonstrated a strong performance. With appropriate analytical and clinical validation and regulatory approval, this assay may be utilized on the widely installed cobas 5800/6800/8800 systems to help meet the growing demand for an HTP *C. auris* assay. The data presented originate from internally conducted studies performed for research purposes. The cobas *C. auris* UC assay is not approved for *in vitro* diagnostic use.

## MATERIALS AND METHODS

### *C. auris* cobas omni Utility Channel assay

The pre-formulated assay specific *C. auris* TaqMan primer and probe pool utilized for these internal research studies were purchased through Integrated DNA Technology (catalog #10017949; Integrated DNA Technologies, Inc. Coralville, IA). This 640 µL oligo pool was utilized, along with the Kit cobas 5800/6800/8800 Utility Channel 192T IVD Cassette (catalog #9052011190; Roche, Branchburg, NJ), to perform the following studies. As per the manufacturer’s instructions, 600 µL of the prepared oligo pool was added to 10 mL of the supplied Master Mix R2 from the UC kit. This was mixed in a user-supplied 50 mL capped tube and then added to the UC cassette as per manufacturer’s instructions. The RFID on the UC cassette was programmed with the cobas *C. auris* UC assay parameters (listed below), and the cassette was loaded into the cobas system for testing.

Our *C. auris* testing utilized a Uniswab with 4.3 mL cobas PCR media (catalog #7958030190, Roche) or 1 mL liquid Amies ESwab (catalog #480C; Copan Diagnostics Inc., Murrieta, CA) for collection devices coupled with an on-machine sample input volume of 400 µL (unless otherwise mentioned) using the UC Analysis Package for “U_Sample with Swab.” The cycling conditions included a three-step pre-PCR cycle of 55°C for 120 s, followed by 60°C for 360 s and 65°C for 240 s. The first measurement (5 cycles) included a two-step PCR cycle of 95°C for 5 s and 55°C for 30 s. The second measurement (45 cycles) included a two-step PCR cycle of 91°C for 5 s and 58°C for 25 s. A Ct and relative fluorescence increase (RFI) value was defined for the *C. auris* target (channel 3) (Ct≤38, RFI ≥ 2.5) and the internal control (channel 5) (Ct≤50, RFI ≥ 2) to report valid and positive sample results. The UC negative control cassette (catalog #7001185190, Roche) was utilized with a Ct of ≥38 and an RFI of ≤2.5 cutoffs to validate each run. Additionally, a positive control (in-house plasmid with the *C. auris* target sequence or commercially available Z485 *C. auris* strain [catalog #0804386; Zeptometrix, Buffalo, NY], diluted 10^4^ CFU/mL in either CPM or liquid Amies) was processed as a sample in each run. Data shown was processed on a cobas 6800 instrument but should be applicable to cobas 5800 and 8800 systems.

### Inclusivity and exclusivity

Inclusivity of the *C. auris* assay was analyzed using internal proprietary *in silico* software and 736 NCBI sequences currently available, covering the existing *C. auris* clades (including all available sixth clade sequences). Over 99% of available inclusive sequences are predicted to have a Ct within five cycles. Additionally, wet lab testing verified inclusivity of representative CDC strains from clades I–V (AR-0387, AR-0381, AR-0383, AR-0385, and AR-1097 from the CDC and Food and Drug Administration Antimicrobial Resistance Isolate Bank Panel *C. auris*) (Centers for Disease Control and Prevention, Atlanta, GA). No clade VI strains were available for wet lab testing. The CDC strains were plated on Sabouraud dextrose agar plates (catalog #210950; BD Difco, Sparks, MD) and grown at 37°C for 48 h. Single colonies were selected into Sabouraud dextrose broth media (catalog #238230, BD Difco) and grown overnight at 37°C, shaken at 225 RPM. Day cultures were then inoculated into fresh media and grown in the same conditions for 6 h. Cultures were spun down for 10 m at 5,000 × *g* to pellet the cells, and the media were removed. The pellet was re-suspended in 3 mL Tris-EDTA (TE) buffer, and the optical density (OD) was measured at 530 nm and normalized to OD = 0.01 (representing 10^5^ CFU/mL). Cell load was confirmed by serial dilution in TE buffer and triplicate plating on Sabouraud dextrose agar. A final concentration of 100 and 10 CFU/mL was created and tested in triplicate using the cobas *C. auris* UC assay as described above using a 400 µL input volume.

Assay exclusivity was determined by testing against an extensive panel of closely related *Candida* spp. (Table 1). Strains analyzed were obtained from the CDC and FDA AR Isolate Bank, American Type Culture Collection (ATCC, Gaithersburg, MD), and United States Department of Agriculture Agricultural Research Service Culture Collection (Peoria, IL). The *C. haemulonii*, *C. duobushaemuli*, *C. sake*, and *C. famata* strains were grown at 30°C, while the remaining strains were grown at 37°C. All exclusive strains were grown in Sabouraud dextrose broth media shaken at 225 RPM with optical density measured at 530 nm as previously stated and adjusted to OD = 1, representing a final concentration of 10^7^ CFU/mL. The CDC AR-383 *C. auris* strain was tested at 10^4^ CFU/mL as a positive control for these experiments. Samples were processed in duplicate using the cobas *C. auris* UC assay as described above using a 400 µL input volume.

### Linearity and negative collection media testing

Commercially available and titered *C. auris* Z485 cells were used to assess assay linearity. Briefly, cells were thawed on ice and serially diluted in CPM to a final concentration ranging from 10^5^ to 10 CFU/mL. Each dilution was tested using the cobas *C. auris* UC assay as described above using a 400 µL input volume.

Non-specific amplification was tested using both liquid Amies and CPM blank samples. In total, 100 replicates of each collection media were tested (for a total of 200 samples) with each sample containing 1 mL. These samples were processed using a 400 µL input volume with the assay conditions stated above along with positive control samples on each plate to verify normal assay performance.

### LoD

Analytical sensitivity was determined in two different clinical backgrounds using the AR-0383 *C. auris* cell line. Day cultures were grown and normalized in TE buffer as previously stated, with CFU counts performed in triplicate to confirm the normalized cells. Subsequently, the normalized cells were spiked into negative *C. auris* axilla/groin or nasal swab matrix obtained from healthy donors using the cobas UniSwab sample collection kits and serially diluted to a final concentration range from 133 to 16 CFU/mL. Each dilution from both clinical backgrounds was tested with 20 replicates using the cobas *C. auris* UC assay as described above using a 400 µL input volume. A probit regression analysis based on a 95% hit rate using SAS JMP statistical software (Corporate-JMP, Cary, NC) was used to calculate the analytical sensitivity (LoD).

### Clinical sample evaluation

Two hundred de-identified remnant composite nasal/axilla/groin ESwab samples from multiple US healthcare facilities were previously analyzed by the Wadsworth Center of New York State Department of Health using the BD MAX Open System *C. auris* assay as part of their clinical surveillance workflow (24). In addition to BD MAX testing, 159 of the 200 samples were also tested using a culture enrichment workflow based on sample growth from Salt Dulcitol Broth (grown at 40°C until turbid) followed by culture on Sabouraud dextrose agar (48–72 h at 40°C) to obtain single colonies for species confirmation based on MALDI-TOF.

The 200 remnant samples were tested using the cobas *C. auris* UC assay in two phases (frozen and fresh samples) primarily comparing the assay results to the reference NAAT standard method with a secondary analysis against the culture enrichment workflow. All remnant clinical samples were tested with the cobas *C. auris* UC assay as described above but using only an input volume of 200 µL (rather than the recommended 400 µL) due to the limited remnant sample volume. The first phase (100 samples) was frozen after initial NAAT and culture enrichment testing (if applicable) at the Wadsworth Center and later thawed before testing with the cobas *C. auris* UC assay. The second phase (100 samples) was processed at the Wadsworth Center but then held at 4°C for 9–22 days before being shipped and processed with the cobas *C. auris* UC assay. Importantly, the reference NAAT results from the BD MAX workflow and culture enrichment results were withheld until the samples had completed cobas testing to prevent any bias.

## SUPPLEMENTAL MATERIAL

Table S1 spectrum.00254-25-s0001.docx Clinical sample results for both NAAT workflows as well as culture enrichment.

ASM does not own the copyrights to Supplemental Material that may be linked to, or accessed through, an article. The authors have granted ASM a non-exclusive, world-wide license to publish the Supplemental Material files. Please contact the corresponding author directly for reuse.
