# Supplementary material for: Evaluation of a high-throughput Candidozyma auris assay for use on the cobas 5800/6800/8800 omni Utility Channel
Source: Microbiol Spectr. 2025 May 16;13(7):e00254-25. doi: 10.1128/spectrum.00254-25 (PMC12211074; doi:10.1128/spectrum.00254-25)
Supplement: Table S1 — Clinical sample results for both NAAT workflows as well as culture enrichment. [file spectrum.00254-25-s0001.docx]

**Supplemental Table 1.** Clinical sample results for both NAAT workflows as well as culture enrichment.

|  |  | **cobas® UC assay** | | **Wadsworth Center Results**  (all samples tested fresh) | | |
| --- | --- | --- | --- | --- | --- | --- |
|  |  |  |  | **BD MAX**^™^ **Workflow** | | **Culture Enrichment Result** |
|  | **Sample #** | **Result** | **CT** | **Result** | **CT** |  |
| **Frozen** | ***Cauris_01*** | **Positive** | **25.9** | **Positive** | **27.1** | ***C. auris*** |
|  | ***Cauris_02*** | Negative | n/a | Negative | n/a | *Not cultured* |
|  | ***Cauris_03*** | Negative | n/a | Negative | n/a | *Not cultured* |
|  | ***Cauris_04*** | Negative | n/a | Negative | n/a | *Not cultured* |
|  | ***Cauris_05*** | Negative | n/a | Negative | n/a | *Not cultured* |
|  | ***Cauris_06*** | Negative | n/a | Negative | n/a | *Not cultured* |
|  | ***Cauris_07*** | Negative | n/a | Negative | n/a | *Not cultured* |
|  | ***Cauris_08*** | Negative | n/a | Negative | n/a | *Not cultured* |
|  | ***Cauris_09*** | Negative | n/a | Negative | n/a | *Not cultured* |
|  | ***Cauris_10*** | Negative | n/a | Negative | n/a | *Not cultured* |
|  | ***Cauris_11*** | Negative | n/a | Negative | n/a | *Not cultured* |
|  | ***Cauris_12*** | Negative | n/a | **Positive** | **32.5** | no growth |
|  | ***Cauris_13*** | Negative | n/a | Negative | n/a | *Not cultured* |
|  | ***Cauris_14*** | **Positive** | **36.6** | **Positive** | **35.4** | no growth |
|  | ***Cauris_15*** | Negative | n/a | Negative | n/a | *Not cultured* |
|  | ***Cauris_16*** | Negative | n/a | Negative | n/a | *Not cultured* |
|  | ***Cauris_17*** | **Positive** | **18.4** | **Positive** | **19.7** | ***C. auris*** |
|  | ***Cauris_18*** | Negative | n/a | **Positive** | **37.5** | no growth |
|  | ***Cauris_19*** | Negative | n/a | Negative | n/a | *Not cultured* |
|  | ***Cauris_20*** | **Positive** | **24.9** | **Positive** | **22.3** | ***C. auris*** |
|  | ***Cauris_21*** | Negative | n/a | Negative | n/a | *Not cultured* |
|  | ***Cauris_22*** | Negative | n/a | Negative | n/a | *Not cultured* |
|  | ***Cauris_23*** | Negative | n/a | Negative | n/a | *Not cultured* |
|  | ***Cauris_24*** | **Positive** | **24.5** | **Positive** | **23.9** | ***C. auris*** |
|  | ***Cauris_25*** | Negative | n/a | Negative | n/a | *Not cultured* |
|  | ***Cauris_26*** | Negative | n/a | Negative | n/a | *Not cultured* |
|  | ***Cauris_27*** | **Positive** | **24.6** | **Positive** | **22.3** | ***C. auris*** |
|  | ***Cauris_28*** | Negative | n/a | Negative | n/a | *Not cultured* |
|  | ***Cauris_29*** | **Positive** | **18.6** | **Positive** | **19.5** | ***C. auris*** |
|  | ***Cauris_30*** | Negative | n/a | Negative | n/a | *Not cultured* |
|  | ***Cauris_31*** | Negative | n/a | Negative | n/a | *Not cultured* |
|  | ***Cauris_32*** | Negative | n/a | Negative | n/a | *Not cultured* |
|  | ***Cauris_33*** | **Positive** | **27.5** | **Positive** | **25.6** | ***C. auris*** |
|  | ***Cauris_34*** | Negative | n/a | Negative | n/a | *Not cultured* |
|  | ***Cauris_35*** | Negative | n/a | **Positive** | **35.0** | no growth |
|  | ***Cauris_36*** | Negative | n/a | Negative | n/a | *Not cultured* |
|  | ***Cauris_37*** | Negative | n/a | Negative | n/a | *Not cultured* |
|  | ***Cauris_38*** | Negative | n/a | Negative | n/a | *Not cultured* |
| **Frozen** | ***Cauris_39*** | **Positive** | **20.1** | **Positive** | **20.3** | ***C. auris*** |
|  | ***Cauris_40*** | Negative | n/a | Negative | n/a | *Not cultured* |
|  | ***Cauris_41*** | **Positive** | **34.0** | **Positive** | **32.9** | E. coli |
|  | ***Cauris_42*** | Negative | n/a | Negative | n/a | *Not cultured* |
|  | ***Cauris_43*** | Negative | n/a | Negative | n/a | *Not cultured* |
|  | ***Cauris_44*** | **Positive** | **31.0** | **Positive** | **22.6** | ***C. auris*** |
|  | ***Cauris_45*** | Negative | n/a | Negative | n/a | *Not cultured* |
|  | ***Cauris_46*** | Negative | n/a | Negative | n/a | *Not cultured* |
|  | ***Cauris_47*** | **Positive** | **29.1** | **Positive** | **28.8** | ***C. auris*** |
|  | ***Cauris_48*** | Negative | n/a | Negative | n/a | *Not cultured* |
|  | ***Cauris_49*** | Negative | n/a | Negative | n/a | *Not cultured* |
|  | ***Cauris_50*** | Negative | n/a | Negative | n/a | *Not cultured* |
|  | ***Cauris_51*** | **Positive** | **22.7** | **Positive** | **21.1** | ***C. auris*** |
|  | ***Cauris_52*** | **Positive** | **25.6** | **Positive** | **23.1** | ***C. auris / E. faecalis*** |
|  | ***Cauris_53*** | Negative | n/a | Negative | **n/a** | *Not cultured* |
|  | ***Cauris_54*** | **Positive** | **23.7** | **Positive** | **20.3** | ***C. auris*** |
|  | ***Cauris_55*** | **Positive** | **29.5** | **Positive** | **31.0** | ***C. auris*** |
|  | ***Cauris_56*** | Negative | n/a | Negative | n/a | *Not cultured* |
|  | ***Cauris_57*** | **Positive** | **23.0** | **Positive** | **22.7** | ***C. auris*** |
|  | ***Cauris_58*** | Negative | n/a | Negative | n/a | *Not cultured* |
|  | ***Cauris_59*** | Negative | n/a | **Positive** | **33.4** | *C. albicans/E. coli* |
|  | ***Cauris_60*** | Negative | n/a | Negative | n/a | *Not cultured* |
|  | ***Cauris_61*** | **Positive** | **33.3** | **Positive** | **30.8** | ***C. auris*** |
|  | ***Cauris_62*** | Negative | n/a | **Positive** | **32.8** | no growth |
|  | ***Cauris_63*** | Negative | n/a | Negative | n/a | *Not cultured* |
|  | ***Cauris_64*** | Negative | n/a | Negative | n/a | *Not cultured* |
|  | ***Cauris_65*** | **Positive** | **22.4** | **Positive** | **23.4** | ***C. auris*** |
|  | ***Cauris_66*** | Negative | n/a | **Positive** | **34.0** | no growth |
|  | ***Cauris_67*** | **Positive** | **25.2** | **Positive** | **25.9** | ***C. auris*** |
|  | ***Cauris_68*** | **Positive** | **22.9** | **Positive** | **23.1** | ***C. auris*** |
|  | ***Cauris_69*** | **Positive** | **28.9** | **Positive** | **26.6** | ***C. auris*** |
|  | ***Cauris_70*** | **Positive** | **22.1** | **Positive** | **22.2** | ***C. auris*** |
|  | ***Cauris_71*** | **Positive** | **23.8** | **Positive** | **23.5** | ***C. auris*** |
|  | ***Cauris_72*** | Negative | n/a | Negative | n/a | No growth |
|  | ***Cauris_73*** | **Positive** | **22.1** | **Positive** | **19.3** | ***C. auris*** |
|  | ***Cauris_74*** | **Positive** | **22.7** | **Positive** | **21.5** | ***C. auris*** |
|  | ***Cauris_75*** | **Positive** | **21.0** | **Positive** | **24.8** | ***C. auris*** |
|  | ***Cauris_76*** | **Positive** | **23.5** | **Positive** | **25.2** | ***C. auris*** |
|  | ***Cauris_77*** | **Positive** | **21.9** | **Positive** | **23.0** | ***C. auris*** |
|  | ***Cauris_78*** | **Positive** | **17.0** | **Positive** | **20.3** | ***C. auris*** |
|  | ***Cauris_79*** | Negative | n/a | Negative | n/a | no growth |
|  | ***Cauris_80*** | **Positive** | **23.5** | **Positive** | **26.4** | ***C. auris*** |
|  | ***Cauris_81*** | **Positive** | **29.3** | **Positive** | **28.4** | ***C. auris*** |
| **Frozen** | ***Cauris_82*** | **Positive** | **33.1** | **Positive** | **31.7** | ***C. auris*** |
|  | ***Cauris_83*** | **Positive** | **22.5** | **Positive** | **24.3** | ***C. auris*** |
|  | ***Cauris_84*** | **Positive** | **23.0** | **Positive** | **20.8** | ***C. auris*** |
|  | ***Cauris_85*** | **Positive** | **27.5** | **Positive** | **27.4** | ***C. auris*** |
|  | ***Cauris_86*** | **Positive** | **20.1** | **Positive** | **19.1** | ***C. auris*** |
|  | ***Cauris_87*** | **Positive** | **17.7** | **Positive** | **22.3** | ***C. auris*** |
|  | ***Cauris_88*** | **Positive** | **22.5** | **Positive** | **21.1** | ***C. auris*** |
|  | ***Cauris_89*** | **Positive** | **21.0** | **Positive** | **23.8** | ***C. auris*** |
|  | ***Cauris_90*** | Negative | n/a | Negative | n/a | no growth |
|  | ***Cauris_91*** | Negative | n/a | Negative | n/a | no growth |
|  | ***Cauris_92*** | Negative | n/a | Negative | n/a | no growth |
|  | ***Cauris_93*** | **Positive** | **23.1** | **Positive** | **22.2** | ***C. auris*** |
|  | ***Cauris_94*** | **Positive** | **24.6** | **Positive** | **23.6** | ***C. auris*** |
|  | ***Cauris_95*** | **Positive** | **19.0** | **Positive** | **18.0** | ***C. auris*** |
|  | ***Cauris_96*** | **Positive** | **19.7** | **Positive** | **23.1** | ***C. auris*** |
|  | ***Cauris_97*** | **Positive** | **20.3** | **Positive** | **21.0** | ***C. auris*** |
|  | ***Cauris_98*** | **Positive** | **16.2** | **Positive** | **16.9** | ***C. auris*** |
|  | ***Cauris_99*** | Negative | n/a | Negative | n/a | no growth |
|  | ***Cauris_100*** | Negative | n/a | Negative | n/a | no growth |
| **Fresh** | ***Cauris_01*** | **Positive** | **23.13** | **Positive** | **22.9** | ***C. auris*** |
|  | ***Cauris_02*** | Negative | n/a | Negative | n/a | no growth |
|  | ***Cauris_03*** | Negative | n/a | Negative | n/a | no growth |
|  | ***Cauris_04*** | **Positive** | **29.93** | **Positive** | **28.0** | ***C. auris*** |
|  | ***Cauris_05*** | **Positive** | **32.16** | **Positive** | **31.2** | no growth |
|  | ***Cauris_06*** | Negative | n/a | Negative | n/a | no growth |
|  | ***Cauris_07*** | Negative | n/a | Negative | n/a | no growth |
|  | ***Cauris_08*** | **Positive** | **36.53** | Negative | n/a | no growth |
|  | ***Cauris_09*** | Negative | n/a | Negative | n/a | no growth |
|  | ***Cauris_10*** | Negative | n/a | Negative | n/a | no growth |
|  | ***Cauris_11*** | **Positive** | **37.27** | **Positive** | **33.1** | no growth |
|  | ***Cauris_12*** | **Positive** | **29.26** | **Positive** | **28.6** | ***C. auris*** |
|  | ***Cauris_13*** | Negative | n/a | Negative | n/a | no growth |
|  | ***Cauris_14*** | **Positive** | **37.95** | Negative | n/a | no growth |
|  | ***Cauris_15*** | Negative | n/a | Negative | n/a | no growth |
|  | ***Cauris_16*** | Negative | n/a | Negative | n/a | no growth |
|  | ***Cauris_17*** | Negative | n/a | Negative | n/a | no growth |
|  | ***Cauris_18*** | **Positive** | **27.31** | **Positive** | **27.9** | ***C. auris*** |
|  | ***Cauris_19*** | Negative | n/a | Negative | n/a | no growth |
|  | ***Cauris_20*** | Negative | n/a | Negative | n/a | no growth |
|  | ***Cauris_21*** | Negative | n/a | Negative | n/a | no growth |
|  | ***Cauris_22*** | Negative | n/a | Negative | n/a | no growth |
|  | ***Cauris_23*** | Negative | n/a | Negative | n/a | no growth |
|  | ***Cauris_24*** | Negative | n/a | Negative | n/a | no growth |
| **Fresh** | ***Cauris_25*** | Negative | n/a | Negative | n/a | no growth |
|  | ***Cauris_26*** | Negative | n/a | Negative | n/a | no growth |
|  | ***Cauris_27*** | **Positive** | **22.21** | **Positive** | **21.8** | ***C. auris*** |
|  | ***Cauris_28*** | Negative | n/a | Negative | n/a | no growth |
|  | ***Cauris_29*** | Negative | n/a | Negative | n/a | no growth |
|  | ***Cauris_30*** | **Positive** | **20.41** | **Positive** | **23.5** | ***C. auris*** |
|  | ***Cauris_31*** | **Positive** | **28.06** | **Positive** | **26.7** | ***C. auris*** |
|  | ***Cauris_32*** | **Positive** | **21.24** | **Positive** | **21.3** | ***C. auris*** |
|  | ***Cauris_33*** | **Positive** | **26.04** | **Positive** | **26.0** | ***C. auris*** |
|  | ***Cauris_34*** | **Positive** | **25.61** | **Positive** | **29.1** | ***C. auris*** |
|  | ***Cauris_35*** | **Positive** | **18.08** | **Positive** | **18.6** | ***C. auris*** |
|  | ***Cauris_36*** | **Positive** | **23.22** | **Positive** | **21.2** | ***C. auris*** |
|  | ***Cauris_37*** | **Positive** | **32.4** | **Positive** | **30.7** | ***C. auris*** |
|  | ***Cauris_38*** | **Positive** | **23.87** | **Positive** | **18.9** | ***C. auris*** |
|  | ***Cauris_39*** | Negative | n/a | Negative | n/a | no growth |
|  | ***Cauris_40*** | **Positive** | **25.08** | **Positive** | **28.9** | ***C. auris*** |
|  | ***Cauris_41*** | Negative | n/a | Negative | n/a | no growth |
|  | ***Cauris_42*** | Negative | n/a | Negative | n/a | no growth |
|  | ***Cauris_43*** | Negative | n/a | Negative | n/a | no growth |
|  | ***Cauris_44*** | Negative | n/a | Negative | n/a | no growth |
|  | ***Cauris_45*** | Negative | n/a | Negative | n/a | no growth |
|  | ***Cauris_46*** | Negative | n/a | Negative | n/a | no growth |
|  | ***Cauris_47*** | Negative | n/a | Negative | n/a | no growth |
|  | ***Cauris_48*** | Negative | n/a | Negative | n/a | no growth |
|  | ***Cauris_49*** | Negative | n/a | Negative | n/a | no growth |
|  | ***Cauris_50*** | **Positive** | **26.87** | **Positive** | **23.3** | ***C. auris*** |
|  | ***Cauris_51*** | Negative | ******* | **Positive** | **30.8** | ***C. auris*** |
|  | ***Cauris_52*** | Negative | n/a | Negative | n/a | no growth |
|  | ***Cauris_53*** | Negative | n/a | Negative | n/a | no growth |
|  | ***Cauris_54*** | **Positive** | **18.22** | **Positive** | **17.5** | ***C. auris*** |
|  | ***Cauris_55*** | Negative | n/a | Negative | n/a | no growth |
|  | ***Cauris_56*** | **Positive** | **27.17** | **Positive** | **23.7** | ***C. auris*** |
|  | ***Cauris_57*** | **Positive** | **31.35** | **Positive** | **33.6** | ***C. auris*** |
|  | ***Cauris_58*** | Negative | n/a | Negative | n/a | *C. parapsilosis* |
|  | ***Cauris_59*** | **Positive** | **23.17** | **Positive** | **22.8** | ***C. auris*** |
|  | ***Cauris_60*** | **Positive** | **30.18** | **Positive** | **32.4** | ***C. auris*** |
|  | ***Cauris_61*** | **Positive** | **20.87** | **Positive** | **22.8** | ***C. auris*** |
|  | ***Cauris_62*** | **Positive** | **21.01** | **Positive** | **20.7** | ***C. auris*** |
|  | ***Cauris_63*** | **Positive** | **29.08** | **Positive** | **26.5** | ***C. auris*** |
|  | ***Cauris_64*** | **Positive** | **19.75** | **Positive** | **21.8** | ***C. auris*** |
|  | ***Cauris_65*** | **Positive** | **28.87** | **Positive** | **27.1** | ***C. auris*** |
|  | ***Cauris_66*** | **Positive** | **26.61** | **Positive** | **26.5** | ***C. auris*** |
|  | ***Cauris_67*** | **Positive** | **22.68** | **Positive** | **21.7** | ***C. auris*** |
| **Fresh** | ***Cauris_68*** | **Positive** | **32.75** | **Positive** | **29.1** | ***C. auris*** |
|  | ***Cauris_69*** | **Positive** | **30.53** | **Positive** | **30.6** | ***C. auris*** |
|  | ***Cauris_70*** | **Positive** | **28.44** | **Positive** | **29.0** | ***C. auris*** |
|  | ***Cauris_71*** | Negative | n/a | Negative | n/a | *C. parapsilosis* |
|  | ***Cauris_72*** | Negative | n/a | Negative | n/a | no growth |
|  | ***Cauris_73*** | Negative | n/a | Negative | n/a | no growth |
|  | ***Cauris_74*** | **Positive** | **27.9** | **Positive** | **27.3** | ***C. auris*** |
|  | ***Cauris_75*** | **Positive** | **28.45** | **Positive** | **27.9** | ***C. auris*** |
|  | ***Cauris_76*** | Negative | n/a | Negative | n/a | no growth |
|  | ***Cauris_77*** | **Positive** | **23.86** | **Positive** | **21.6** | ***C. auris*** |
|  | ***Cauris_78*** | Negative | n/a | Negative | n/a | no growth |
|  | ***Cauris_79*** | **Positive** | **27.89** | **Positive** | **27.7** | ***C. auris*** |
|  | ***Cauris_80*** | Negative | n/a | Negative | n/a | no growth |
|  | ***Cauris_81*** | Negative | n/a | Negative | n/a | no growth |
|  | ***Cauris_82*** | **Positive** | **34.12** | **Positive** | **32.5** | ***C. auris*** |
|  | ***Cauris_83*** | Negative | n/a | Negative | n/a | no growth |
|  | ***Cauris_84*** | **Positive** | **15.99** | **Positive** | **20.6** | ***C. auris*** |
|  | ***Cauris_85*** | **Positive** | **22** | **Positive** | **22.7** | ***C. auris*** |
|  | ***Cauris_86*** | **Positive** | **22.67** | **Positive** | **28.2** | ***C. auris*** |
|  | ***Cauris_87*** | Negative | n/a | Negative | n/a | no growth |
|  | ***Cauris_88*** | Negative | n/a | Negative | n/a | no growth |
|  | ***Cauris_89*** | Negative | *>38*** | Negative | n/a | no growth |
|  | ***Cauris_90*** | **Positive** | **21.01** | **Positive** | **21.5** | ***C. auris*** |
|  | ***Cauris_91*** | **Positive** | **19.05** | **Positive** | **19.4** | ***C. auris*** |
|  | ***Cauris_92*** | **Positive** | **35.43** | **Positive** | **35.8** | no growth |
|  | ***Cauris_93*** | Negative | *>38*** | **Positive** | **34.9** | ***C. auris*** |
|  | ***Cauris_94*** | Negative | n/a | Negative | n/a | *C. metapsilosis* |
|  | ***Cauris_95*** | Negative | >38** | **Positive** | **36.6** | no growth |
|  | ***Cauris_96*** | Negative | n/a | Negative | n/a | no growth |
|  | ***Cauris_97*** | Negative | n/a | Negative | n/a | no growth |
|  | ***Cauris_98*** | Negative | n/a | Negative | n/a | no growth |
|  | ***Cauris_99*** | Negative | n/a | Negative | n/a | no growth |
|  | ***Cauris_100*** | Negative | n/a | **Positive** | **36.9** | no growth |

** Fresh, Sample 51 was not processed with cobas^®^ UC assay due to insufficient volume and excluded from the analysis*

***Fresh, Samples 89, 93 and 95 called negative due to late Ct passed Ct cutoff*
